# Musculoskeletal Modeling of the Wrist via a Multi Body Simulation

**DOI:** 10.3390/life12040581

**Published:** 2022-04-14

**Authors:** Jörg Eschweiler, Maximilian Praster, Valentin Quack, Roman Michalik, Frank Hildebrand, Björn Rath, Filippo Migliorini

**Affiliations:** 1 Department of Orthopaedics, Trauma and Reconstructive Surgery, RWTH Aachen University Hospital, 52074 Aachen, Germany; mpraster@ukaachen.de (M.P.); vquack@ukaachen.de (V.Q.); rmichalik@ukaachen.de (R.M.); fhildebrand@ukaachen.de (F.H.); fmigliorini@ukaachen.de (F.M.); 2 Department of Orthopaedic Surgery, Klinikum Wels-Grieskirchen, 4600 Wels, Austria; bjoern.rath@klinikum-wegr.at; 3 Department of Orthopaedic and Trauma Surgery, Eifelklinik St. Brigida, 52152 Simmerath, Germany

**Keywords:** MBS, biomechanics, modeling, total wrist arthroplasty

## Abstract

In this study, three different musculoskeletal modeling approaches were compared to each other. The objective was to show the possibilities in the case of a simple mechanical model of the wrist, using a simple multi-body-simulation (MBS) model, and using a more complex and patient-specific adaptable wrist joint MBS model. Musculoskeletal modeling could be a useful alternative, which can be practiced as a non-invasive approach to investigate body motion and internal loads in a wide range of conditions. The goal of this study was the introduction of computer-based modelling of the physiological wrist with (MBS-) models focused on the muscle and joint forces acting on the wrist.

## 1. Introduction

To treat wrist injuries, especially for total wrist arthroplasty (TWA) or degenerative changes, the understanding of the biomechanics and the full function of the wrist is essential. A healthy wrist is crucial for a complete hand function [1]. A deep knowledge of wrist contact forces is fundamental for different reasons: evaluating the effects of treatment, designing better treatments (e.g., implants, rehabilitation regimens), optimizing performance, and obtaining clues to the pathogenesis of the disease [2].

The wrist joint consists of the distal ends of the radius and ulna, eight carpal bones, and interaction with the proximal bases of the five metacarpal bones. The proximal carpal row is described as an intercalated segment [3,4,5,6]. The nearly rigid ligamentous connection of the distal carpal row to the basis of the metacarpal bones allows us to consider the distal row functionally as part of a unit that moves in response to the muscle forces of the forearm [4]. There is coming along with the rigid connection a lack of motion between these bones [4]. The cohesion of the wrist is ensured by the shape of the carpal bones and over 30 ligaments [7].

The wrist motion take place in two main planes: the flexion/extension plane and the radial/ulnar duction plane. A more complex motion, the circumduction motion of the wrist, could be seen as a combination of both planes. Circumduction is the movement of the wrist in which the motion trajectory of the hand describes a circle. It is a combination of flexion, adduction, extension, and abduction of the wrist. The lacked knowledge about what was normal wrist motion and how much wrist motion was utilized in performing activities of daily living (ADLs) is important to know because of the surgical reconstruction and rehabilitation [8]. Functional motion arcs for activities of daily living are 5–10° of flexion to 30–35° extension, and 10° of radial deviation to 15° of ulnar deviation [9].

Musculoskeletal modeling offers an insight into the biomechanics of the healthy and pathological wrist. Muscle forces are the main reason e.g., the joint loads and for resulting endoprosthesis loads, respectively. Producing muscle forces acting on the wrist requires the coordinated action of many muscles, most of them originate in the forearm, cross the wrist, and insert into the fingers [1]. Direct measurement of joint loads and muscle forces is not possible in a normal clinical setting [10].

Detailed, patient-specific information about the forces acting in vivo on and in the wrist when fulfilling activities of the daily life, as well as information on how these forces can be changed via correction of the bony anatomy are not available to the surgeon in clinical routine. The forces acting on the joint or the prosthesis and the way in which transfer this load to the bone also play an essential role in the long-term success of the artificial joint replacement (e.g., function, wear).

Estimation of individual muscle and especially of the resulting joint forces during human motion can provide an insight into neural control and tissue loading. This can contribute to improved diagnosis and management of e.g., neurological or orthopaedic conditions like in the case of TWA [10]. Methods like musculoskeletal and biomechanical modeling, respectively, should therefore be considered [10]. Additionally, musculoskeletal computer models have the possibility to analyze non-invasive the biomechanical consequences of surgical interventions, like TWA, or tendon transfers [1,11]. With such an approach, it would be possible to simulate interventions preoperatively and quantify the outcome. For example, in the case there is a rupture of the scapholunate ligament (SLL), the connection between the scaphoid and lunate bone becomes unstable. The more unstable it becomes, the larger the damaged ligament portion. The SLL is considered the primary stabilizer of the scapholunate joint [12,13]. The evolution of the SLL-lesion and the absence of efficacious treatment have promoted the experimental studies of kinetics and kinematics of the carpus in order to improve the knowledge of carpal instability and look for alternative treatments [14]. Here, models could be a helpful tool that allow to study complex areas as a first step, giving an idea of the necessity of clinical investigation. Therefore, the development of a biomechanical model of the wrist can be an option in exploring the biomechanical behavior of this complex joint system.

In the context of biomechanical modeling of natural joint systems, there exist beside the classical technical mechanical description two traditional simulation approaches to create a computational model of a physiological joint system: firstly, the finite element analysis (FEA)/finite element method (FEM) for stress and strain analysis, and secondly, the multi-body simulation (MBS) approach related to kinematics, kinetics. With the FEA it is possible to address contact problems, but this is not always applicable for complex systems like the wrist joint [15]. The FEA models are valuable tools to create an idea of the behavior of any structure [14]. Especially, wrist modeling via MBS technique can be found in different studies. Currently, there exist different models simulating wrist biomechanics described in the literature [11,16,17,18,19,20,21,22,23].

The objective of this study was to show how mathematical predictions using various modeling techniques can be useful for the acquisition of information which are otherwise not available. It was already opined that computational models will become fundamental tools in biomechanics to address future research questions and clinical applications [24,25]. Different modeling techniques with increasing complexity were applied to a clinical parameter like the muscle load. A comparison of the simulation outcomes of a simple MBS model to a more complex wrist joint MBS-model vs. a simple mechanical model of the wrist were realized. The intention was to introduce the dynamic computer-based modeling of the intact wrist joint with MBS models focused on the muscle and joint forces acting on the wrist. Literature information were used to validate the models and simulation results. The question to answer was: “How complex and personalized must a model be to implement it into the clinical workflow?”

## 2. Material and Methods

### 2.1. General Approach of Modelling

Musculoskeletal models of the wrist joint were developed to examine the muscle forces acting on the wrist joint during flexion-extension (FE) (80°–0°–60°) and radial-ulnar-deviation (RUD) (20°–0°–40°). There was a simple mechanical model of the wrist joint developed based on simple mechanical equations. Furthermore, the implemented simple MBS model of the AnyBody Modeling System (AMS) (AnyBody Technology A/S, Aalborg, Denmark, Version 7) was used to evaluate the forces acting on the wrist. Additionally, a more complex wrist joint MBS model developed by our research group was used to calculate the load of the wrist joint [16,17].

### 2.2. Simple Mechanical Wrist Model

**Figure 1:**
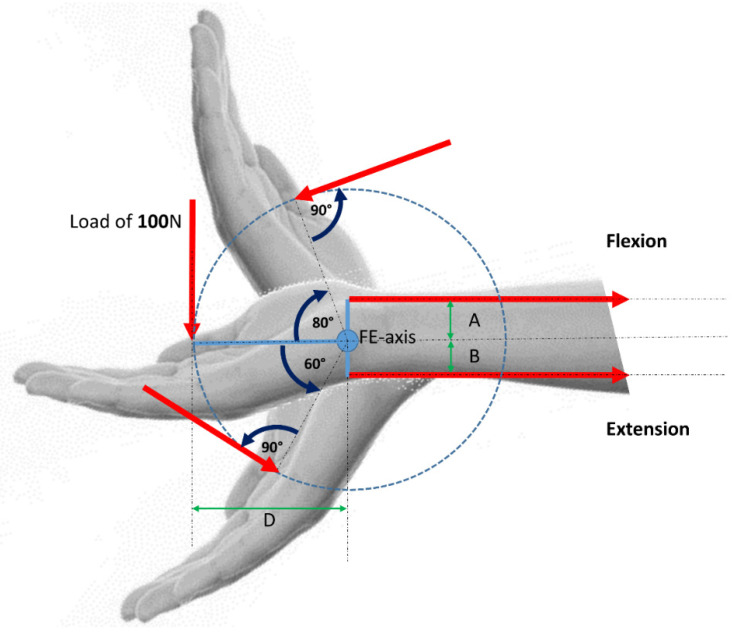
Motion of the wrist in the FE-plane.

**Figure 2:**
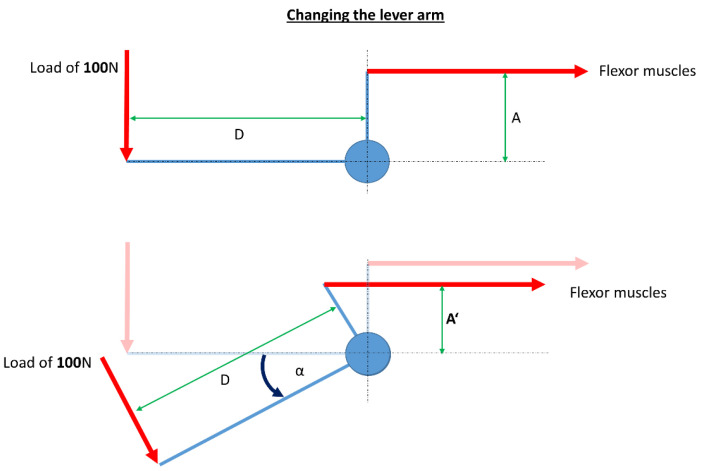
Free body diagram for the change of the lever arm of the summarized flexor muscles in case of wrist extension with a load of 100 N applied to the third metacarpal bone. D is the lever arm of the applied load, (A) is the lever arm of the summarized flexor muscles, and (A′) is the new lever arm depending on the angle of extension of the wrist.

Modeling of the wrist joint with a simplified mechanical model in a static situation is a very easy possibility (Figure 1 and Figure 2), and this approach is well established for other joints e.g., by Pauwels [26] or Brand [2,27] for the hip joint. Especially Pauwels showed that with this approach it was possible to calculate the resulting hip joint force with a high accuracy compared to in vivo measurements [28,29,30].

The simple mechanical description of the wrist includes the summarized flexor muscles, the summarized extensor muscles, and the geometrical relationships.

For example, in case of calculating the muscle forces of the summarized flexor muscles along the free body diagram shown in Figure 2 you have to solve the following equation:

(1) $$F\cdot D=F_{M}\cdot A$$

where F is the external load in [N], D is the lever arm of the external load, F_M_ is the summarized muscle force of the flexor muscles, and A is the lever arm of all flexor muscles.

To calculate F_M_, F_M_ must be separated:

(2) $$F_{M}=\frac{F\cdot D}{A}$$

The change of the lever arm depends on the angle between the neutral position and the current position (Figure 2). It can be calculated with:

(3) A′=A·cosα

**Figure 3:**
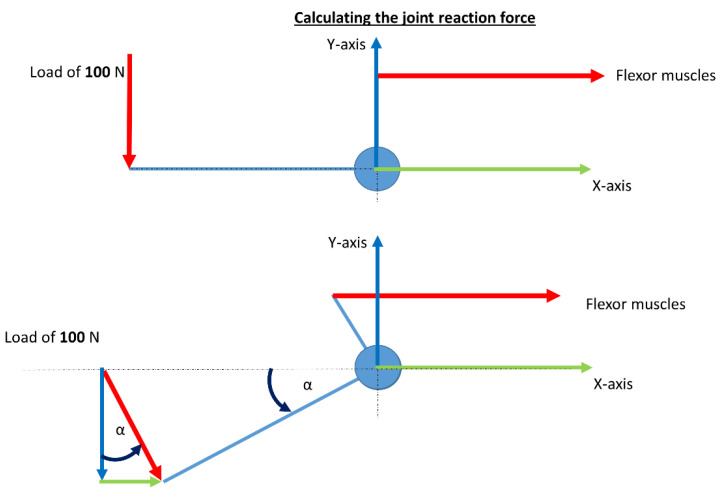
Calculating the joint reaction force of the wrist joint concerning the position of itself with an applied force of 100 N during wrist extension.

To calculate the resulting joint reaction force, you have to summarize the forces in the x- and y-direction separately. For the x-direction you have to solve (see free body diagram Figure 3):

(4) $$F_{X}=\sum^{\;}F_{X1}+F_{X2}+\cdots +F_{\mathrm{Xn}}=0$$

within this case only one external force F_E_ applied:

(5) $$F_{X}=F_{E}\cdot \sin\alpha$$

where F_E_ is 100 N, and α is the angle between F_E_ and the vertical. F_E_ is applied to the third metacarpal bone.

For the y-direction you have to solve:

(6) $$F_{Y}=\sum^{\;}F_{Y1}+F_{Y2}+\cdots +F_{\mathrm{Yn}}=0$$

within this case also here is just one external force F_E_ applied:

(7) $$F_{Y}=F_{E}\cdot \cos\alpha$$

For the resulting external joint reaction force you have to solve:

(8) $$F_{R}=\sqrt{F_{X}^{2}+F_{Y}^{2}}$$

The muscle forces are separately calculated and have to be considered for the complete joint load.

### 2.3. Musculoskeletal Model of the Standard Wrist Developed in AnyBody

**Figure 4:**
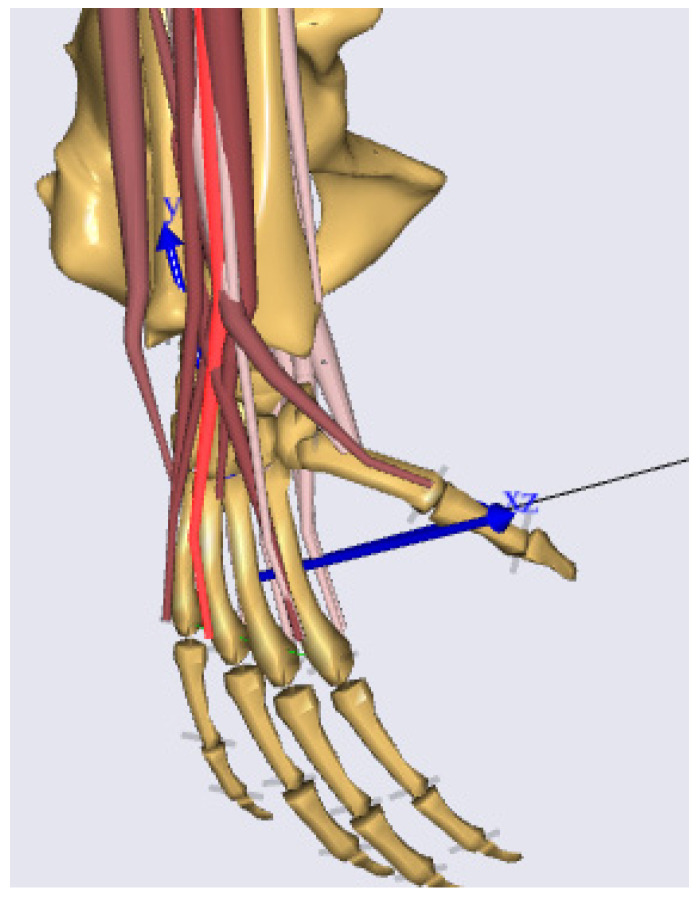
Wrist model of the AnyBody simulation system from a lateral view.

The wrist simulation was performed with the standard wrist model implemented in the AMS (Figure 4). The AMS is a powerful tool that provides both the possibility of developing original models and using in the repository included musculoskeletal models for modeling. The AMS enables inverse dynamic modeling and an additional approach called force-dependent kinematic (FDK). The FDK approach could be taken for the modeling of free form surface interaction of rigid bodies [31,32].

The standard MBS wrist model includes the two forearm bones radius and ulna, the wrist itself approximated as a Cardan joint with two perpendicular rotation axes, and the five fingers (Figure 4). In the end, the FE of the wrist was represented as a revolute joint to account for the motion of the radiocarpal joint. The motion of RUD was also represented as a revolute joint. The MBS model enables simulation of the wrist motion in two planes (FE and RUD).

### 2.4. Musculoskeletal Model of a Complex Wrist Developed in AnyBody

**Figure 5:**
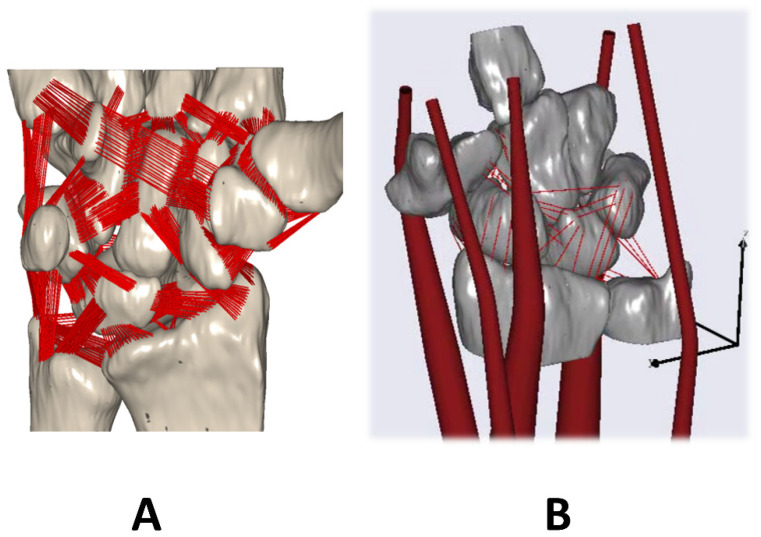
(**A**) Complex wrist model developed in the AnyBody simulation including the ligamentous apparatus. (**B**) Same model with muscles (adapted based on [16,17]).

The implementation of the complex and individual adaptable MBS model of the wrist was performed also with the AMS (V6) (Figure 5) [16,17]. The MBS model enables the simulation of individual (bony) motion and predicts trends in the case of changing kinematics. The model includes the individual wrist bone geometries, the kinematics of the different joints, and the lines of action of the five main forearm muscles acting on the wrist (extensor carpi ulnaris (ECU), extensor carpi radialis brevis (ECRB), extensor carpi radialis longus (ECRL), flexor carpi radialis (FCR), and flexor carpi ulnaris (FCU)).

We did a FE simulation starting for the flexion from 0° up to 60° and back to 0°, similarly for wrist extension [17]. RUD motion was done from 0° up to 40° and back to 0° for ulnar deviation, and radial deviation from 0° to 20° and returned to 0° [17].

For the force calculation, we implemented the FDK approach [31]. The FDK approach assumes a quasi-static force equilibrium between all the acting forces in the model, and it computes the displacements (denoted as α_s_) [31]. Computationally, α_s_ is calculated by introducing a kinematic driver equation in a classical inverse dynamic analysis model to obtain a kinematical determinate system for a given α_s_ [31]:

(9) $$\Phi_{s}\left(q,t\right)-\alpha_{s}=0$$

The function $\Phi_{s}$(q, t) of the model coordinates, q, and time, t, computes the FDK directions [31]. We assumed in our case that the time derivatives of α_s_ are 0 (we obtain a quasi-static solution). We applied an FDK reaction forces F_s_ load in the forearm direction of 140 N.

Now, all of the forces (muscle forces, joint reaction forces, and including the FDK reaction forces) required to balance the MBS model for a given α_s_ can be calculated. All the forces in the model will be balanced via the desired FDK displacements, that is, where the FDK reaction forces are 0. These contact forces depending on the relative deformation between the wrist and forearm, as well as on the contacting surface properties, such as the pressure module. In our case, we used for simulation two different pressure modules (PM): 0.6 × 10^10^ N/m^3^ and 1.2 × 10^10^ N/m^3^.

## 3. Results

### 3.1. Simple Mechanical Wrist Model

**Figure 6:**
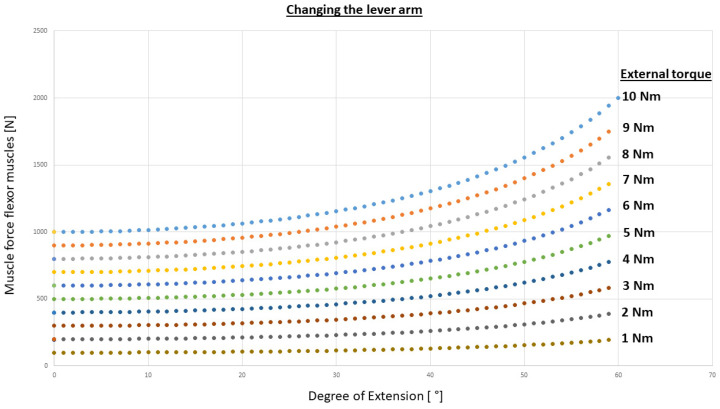
Results of the summarized flexor muscle forces for different external torques concerning the extension angle of the wrist. This is the same for the summarized flexor muscles as for the summarized extensor muscles for the movements during FE.

First, the behavior is investigated for the simple mechanical wrist model with a variation of the lever arm as shown in Figure 2. The effect regarding the resulting global muscle force with simultaneous increase of the extension angle can be seen in Figure 6.

**Figure 7:**
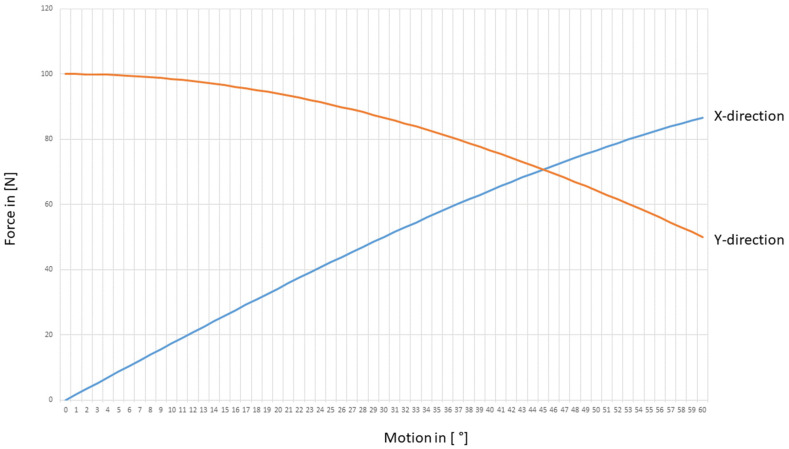
Resulting external part of the joint forces separated in x- and y-direction concerning the motion angle of the wrist. These are just the resulting joint forces based on the external load of 100 N applied to the third metacarpal. The influence of the muscle forces is still missing.

The distribution of the joint forces in the spatial directions x and y under a constant load of 100 N can be found in Figure 7.

### 3.2. Musculoskeletal Standard Model of the Wrist in AnyBody

**Figure 8:**
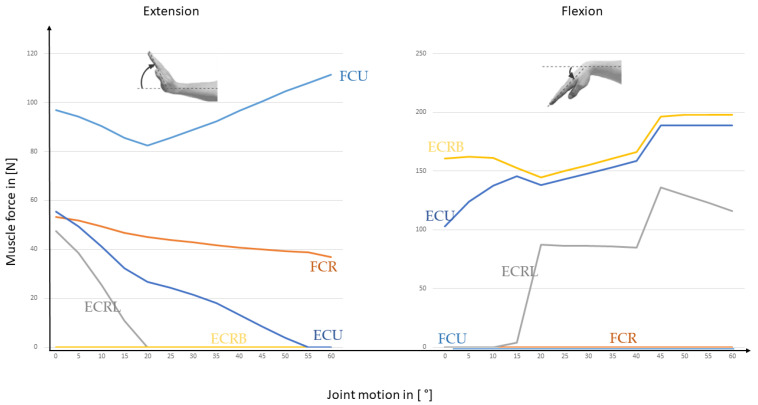
This figure shows the resulting muscle forces during FE motion with an external load of 100 N applied to the third metacarpal.

**Figure 9:**
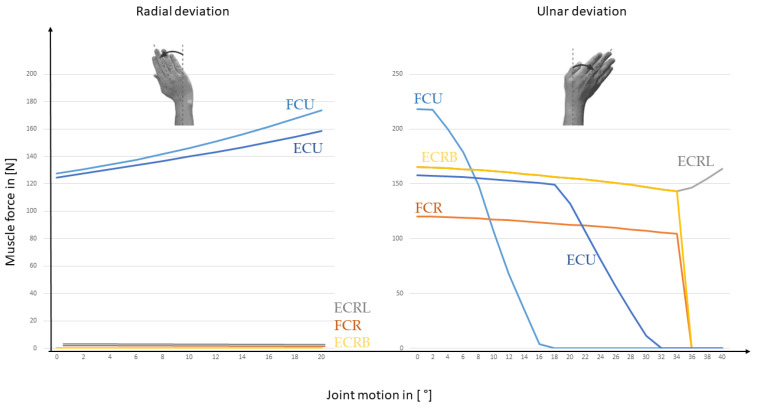
The figure shows the resulting muscle forces during RUD motion with an external load of 100 N applied to the third metacarpal.

For a constant load of 100 N, the results for the FE are represented in Figure 8, while the RUD is shown in Figure 9. Instead of one global muscle force as in the standard mechanical model, now the different muscles of the standard wrist joint model can be presented.

**Figure 10:**
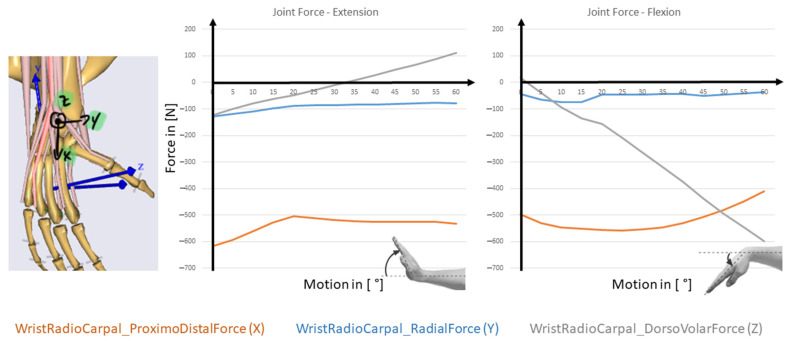
Resulting joint forces during FE motion with an external load of 100 N applied to the third metacarpal.

**Figure 11:**
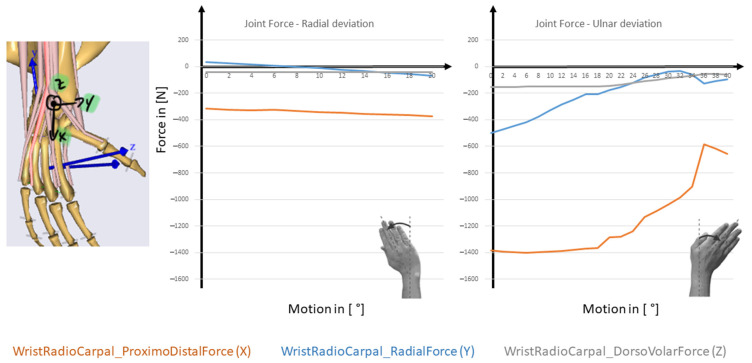
Resulting joint forces during RUD motion with an external load of 100 N applied to the third metacarpal.

The joint reaction forces can now be calculated in the three space directions, that is featured in Figure 10 for FE and in Figure 11 for RUD.

### 3.3. Musculoskeletal Model of the Complex Wrist Developed in AnyBody

**Figure 12:**
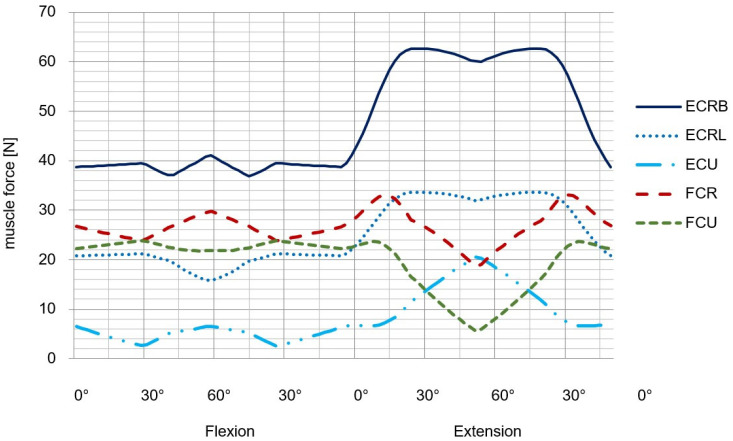
Muscle forces during FE-simulation with an applied load of 100 N to the third metacarpal bone (modified after [16]).

**Figure 13:**
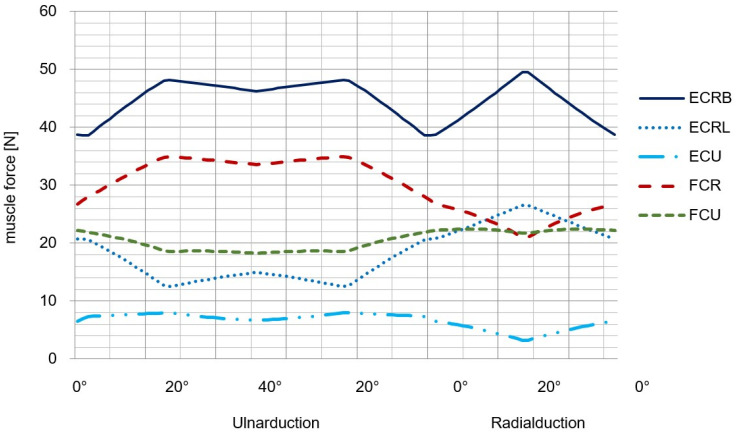
Muscle forces during RUD-simulation with an applied load of 100 N to the third metacarpal bone (modified after [16]).

The last step is now to compare the results obtained in the previous ones with a complex musculoskeletal model of the wrist joint especially developed for therapy planning [16,17]. The five resulting muscle forces for the FE movement is represented in Figure 12 and for the RUD movement in Figure 13.

With the complex wrist model, it’s also possible to get the resulting contact force in the wrist joint. For the load of 140 N it can be found in Table 1.

### 3.4. Data for Validation of the Muscle Forces

For a validation of our results and to evaluate the simulation quality, we took information from the literature from Debottis et al. [33]. The determination of the model properties (e.g., muscle attachment points, wrapping surfaces, bone lengths, joint centers, etc.) is typically based on reference values from anatomic and cadaver studies. The validation values for the different muscle forces are listed in Table 2 and Table 3 and were taken from [33].

## 4. Discussion

The wrist is a complex composition of different structures with different behaviors: bones, soft tissue, and physiological carpal mechanics rely on the interactions between the ligaments and bone geometry [20]. Mathematical techniques for the computation of joint forces provide potentially valuable and unique information regarding the effects of interventions upon joint forces [2]. We used three constructed anatomically approximated musculoskeletal wrist models to biomechanically analyze the wrist during FE and RUD motion using mechanical, inverse dynamic, and FDK calculations. Because of the complexity of the wrist, the calculation of muscle and joint forces is a challenge in the field of biomechanics. Validated models can be used e.g., for a parametrical investigation in the way in which surgical alterations of wrist geometry has an influence on joint forces while varying only one parameter while keeping all other variables constant. In particular, the modeling and simulation can reduce personnel and material require ments for experiments with human material or samples of animal origin [25]. Simulations allow considerations that cannot be justified in animal experiments or clinical trials due to ethical guidelines [25].

### 4.1. Simple Mechanical Model

The major advantages of simple (mathematical) modeling include the capacity to study many normal subjects more or less relatively inexpensively. Using a simple mechanical model for analyzing the motion of the wrist joint and the in this case the resulting muscle and joint force leads to an over prediction of the forces, also the non-linear behavior of the muscles are neglected. In our case, we calculated muscle force up to 2000 N (for a torque of 10 Nm) (Figure 6). This is much higher than in comparison to the MBS models. The results depend on the correct estimation of the lever arm of the FE and RUD involved muscles. This approach of using mathematical models for simulation has the enormous advantage that one can parametrically alter a single variable.

### 4.2. AMS standard MBS Wrist Model

One advantage of the inverse dynamic approach is that the muscle forces can be estimated for all the muscles involved in the motion. The model allows also to change the material law for the muscles so that the nonlinear behavior of the muscles could be addressed. Besides that a personalized model of the different muscles is possible. We focused on the main muscles for FE and RUD. The simulation includes the forces for ECRB, ECRL, FCU, FCR, and ECU. With this approach, it is possible to differentiate between the load of the included muscles. The model allows to get fast results for the involved muscles and a parameter study for that is easy to implement. One disadvantage is, that soft tissue is completely neglected, and with that, the complex kinematics can’t be reproduced.

### 4.3. Complex MBS Wrist Model

In this case, the wrist model simulated joint function and motion behaviour as dictated by articular surface anatomy, ligamentous constraints, and muscle loading. In comparison to the literature, the simulation results of the complex MBS wrist model show similar trends compared to the literature data e.g., from [33]. The simulation results of the muscle forces for ECRB, ECRL, FCU, and FCR are higher than the experimental evaluated results from [33]. In the case of the ECU, they are lower than the experimental results taken from [33] for comparison. The reason for that could be the assumptions made for the maximal muscle force and the muscle length. The data for the calculation of muscle force and length were taken from the literature [17]. The level of agreement of our results may also suggest that the criteria used for solving the muscle recruitment problem, as well as the muscle modeling and strength scaling applied, was able to approximate the particular MS biomechanics for this specific subject to a great extent. This could lead to an overestimation of the simulation results.

To evaluate the model’s sensitivity to the PM chosen in the FDK algorithm, the PM was varied up.

Due to the FDK method’s capability to compute internal forces through quasi-static force equilibrium, it opens up new possibilities in the detailed modelling of joints in musculoskeletal models [32]. This enables the analysis of non-conforming joints under the influence of muscle forces [31].

### 4.4. Limitations

There exist still some limitation. One huge problem is the validation of detected muscle forces with computational/simulation approaches. The fact is that there exists no opportunity to experimental validate the results in its entirety. Studies of muscle force predictions usually compare muscle loading or activation patterns against electromyography (EMG) data as an estimate of validity. In the case of cadaver experiments, the active behavior of the muscle is missing, and only the static/passive characteristics of the soft tissue are considerable. Also, muscle scaling is currently not possible. Three-dimensional models avoid many critical limitations but, like two-dimensional (mechanical) models, are still dependent on assumptions, such as choice of optimization criteria and methods of modelling muscles.

In the case of the complex MBS model, ligaments were modeled as multi one-dimensional nonlinear elastic springs, wrapping around geometrical shapes for preventing bone and implant penetration. This approach allowed a large model simplification. The problem is that it could not mirror the complex stress deformation characteristic of ligament tissue in 3D.

### 4.5. Examples of Possible Use Cases

To underline the possibilities using biomechanical modeling in the clinical workflow, here are some examples:

1. It is possible to show the change of the resulting joint load in case of osteotomies. Via simulation the joint load could be calculated (preoperatively) for different surgical scenarios, and the surgeon could directly see which influence the surgical intervention will have (e.g., [34]).
2. You can prove the quality of surgery. In the case you want to decrease the joint load postoperatively in comparison to the preoperative situation, you can investigate this via model calculation [35,36].
3. You can investigate the influence changing the degrees of freedom (DOF) of a joint. In the case of e.g., a four corner fusion in the wrist you can visualize the new kinematic with reduced DOF and calculate additionally the resulting joint load [37]. Also the load on the implant itself could be calculated.

There exist many more examples for a successful use of modeling and simulation methods in the context of therapy planning. The bottle neck is the implementation of such models and techniques in today’s clinical workflows because of the cost and time pressure, respectively.

### 4.6. Examples of Validation Possibilities

For validation of a modelling and simulation approach there exist different possibilities:

1. For example, there exist checklist and methodology for verification and validation focused on FEA models [25].
2. For the huge joints in the human body (knee, hip, shoulder, and spine) the online database Orthoload (www.orthoload.com, accessed on 13 April 2022) is a perfect approach to use their information to validate own models. Furthermore, kinematic investigation is an alternative method to use the acquired motion trajectories for model validation [38].
3. Also the methods like e.g., sensitivity studies (A sensitivity study quantifies how the uncertainty in the output of a model can be divided and allocated to different sources of uncertainty in its inputs), can be a powerful tool to get an impression of the validity of the model [24].

An overview of verification and validation approaches could be found e.g., in Henninger et al. [24].

In the case of wrist joint biomechanics there exist also some explorative and innovative sources and methods for research and validation of models for the wrist joint [39,40,41,42].

### 4.7. Ongoing Work in the Case of the Complex MBS Model

Ongoing research will address a more detailed representation of the ligamentous restraint in the FDK wrist model. EMG to validate muscle activations, muscle force production, and EMG signal transfer to the modelling approach will be implemented in the future. Also, the extent of forearm muscle co-contractions has to be evaluated. The simultaneous activations of antagonistic muscles can result in increased joint forces [43].

## 5. Conclusions

The modeling approach can be an effective tool in exploring the biomechanical behavior of musculoskeletal systems. The MBS simulation for practical applications in wrist joint surgery, adapted to the individual patient, holds significant promise and enormous challenges. Our objective was to show and understand the existing possibilities to acquire information which are not available in the normal clinical workflow. This powerful advantage cannot be achieved by any other current approach.

The modeling approach is subject to many assumptions. Musculoskeletal modeling should be able to integrate patient-specific parameters and undergo thorough validation before their introduction into clinical practice. If the modeling function of detecting joint and muscle forces is validated, it will also offer a basis for quantifying the effects of ligamentous instabilities, posttraumatic misalignments, and various types of surgical procedures on the wrist joint.

However, trends are likely less affected than absolute values in the case of using a modeling approach, and, there exist reasons to believe that some mathematical estimates are close to reality [27]. However, (complex and personalized) models can inform mission critical applications, such as design of implants and can provide insight into function following medical procedures [44].

The question we want to answer was: “How complex and personalized must a model be to implement it into the clinical workflow?” The answer for that is: “It depends!” In the end, it depends on what you want to know and how accurate it must be. For determining only trends, a simple mechanical model which mirrors the trends relatively is enough. If you want to know more e.g., single muscle forces, you have to increase the complexity from 2D to 3D with more details. A model, i.e., one with no particular personalization, is appropriate when studying e.g., simple biomechanical (musculoskeletal) phenomena decoupled from the individual/group, investigating simple external biomechanical quantities (e.g., in the case of gait analysis spatiotemporal parameters such as locomotion speed, cadence, etc.) which are not sensitive to model personalization. In the case modeling of specific musculoskeletal function of specific individuals/groups the model should properly represent their unique anatomy and physiology [44].

## Figures and Tables

**Table 1:** Resulting joint forces for two different pressure modules in the case of an applied force of 140 N (according to [16,17]). PM: pressure module.

	PM:	PM:
**Contact Matching**	**0.6 × 10^10^ N/m^3^**	**1.2 × 10^10^ N/m^3^**
	**Resulting Contact**	**Resulting Contact**
	**Force (N)**	**Force (N)**
**Scaphoid-Radius**	124.0	185.9
**Lunate-Radius**	49.0	66.8
**Lunate-Ulna**	5.5	9.4
**F_R_**	178.5	262.1

**Table 2:** Average **peak muscle force** [N], in brackets are presented the standard deviation (SD)) during the 4th cycle of each wrist motion (ECRB, ECRL, FCU, (taken from [33])).

Wrist Motion	ECU	ECRB	ECRL	FCR	FCU
FE	54 (15)	26 (7)	60 (22)	32 (12)	33 (5)
RUD	34 (9)	16 (7)	51 (9)	12 (7)	34 (12)

**Table 3:** Average mean muscle force [N], in brackets are presented the standard deviation (SD)) during the 4th cycle of each wrist motion (ECRB, ECRL, FCU (taken from [33])).

Wrist Motion	ECU	ECRB	ECRL	FCR	FCU
FE	23 (2)	11 (6)	25 (6)	17 (3)	15 (5)
RUD	21 (4)	5 (3)	27 (3)	6 (4)	18 (3)
